# Organ and Tissue-Specific Localisation of Selected Cell Wall Epitopes in the Zygotic Embryo of *Brachypodium distachyon*

**DOI:** 10.3390/ijms19030725

**Published:** 2018-03-03

**Authors:** Alexander Betekhtin, Anna Milewska-Hendel, Joanna Lusinska, Lukasz Chajec, Ewa Kurczynska, Robert Hasterok

**Affiliations:** 1 Department of Plant Anatomy and Cytology, Faculty of Biology and Environmental Protection, University of Silesia in Katowice, 28 Jagiellonska Street, 40-032 Katowice, Poland; joanna.lusinska@wp.pl (J.L.); robert.hasterok@us.edu.pl (R.H.); 2 Department of Cell Biology, Faculty of Biology and Environmental Protection, University of Silesia in Katowice, 28 Jagiellonska Street, 40-032 Katowice, Poland; anna.milewska@us.edu.pl (A.M.-H.); ewa.kurczynska@us.edu.pl (E.K.); 3 Department of Animal Histology and Embryology, Faculty of Biology and Environmental Protection, University of Silesia in Katowice, 28 Jagiellonska Street, 40-032 Katowice, Poland; lukasz.chajec@us.edu.pl

**Keywords:** Brachypodium, cell wall, nucleus, zygotic embryo

## Abstract

The plant cell wall shows a great diversity regarding its chemical composition, which may vary significantly even during different developmental stages. In this study, we analysed the distribution of several cell wall epitopes in embryos of *Brachypodium distachyon* (Brachypodium). We also described the variations in the nucleus shape and the number of nucleoli that occurred in some embryo cells. The use of transmission electron microscopy, and histological and immunolocalisation techniques permitted the distribution of selected arabinogalactan proteins, extensins, pectins, and hemicelluloses on the embryo surface, internal cell compartments, and in the context of the cell wall ultrastructure to be demonstrated. We revealed that the majority of arabinogalactan proteins and extensins were distributed on the cell surface and that pectins were the main component of the seed coat and other parts, such as the mesocotyl cell walls and the radicula. Hemicelluloses were localised in the cell wall and outside of the radicula protodermis, respectively. The specific arrangement of those components may indicate their significance during embryo development and seed germination, thus suggesting the importance of their protective functions. Despite the differences in the cell wall composition, we found that some of the antibodies can be used as markers to identify specific cells and the parts of the developing Brachypodium embryo.

## 1. Introduction

The cell wall is a protective layer that is located around the cell membrane, which is found in plant, fungi, bacteria, and archaea cells. The structure and functions of the cell wall were interconnected during the process of evolution in spite of the fact that its chemical structure, and to a lesser extent, its functions are diverse in different groups of organisms. In plants, its most fundamental function is to provide structural support and protection to the cell with some additional specific functions that may occur during plant development and cell differentiation [1].

The plant cell wall is a dynamic and highly specialised network that is formed by a heterogeneous mixture of cellulose, hemicelluloses, pectins and, to some extent, proteins and phenolic compounds. The cell wall that is formed during cell division is called the primary cell wall. In many plants, as/after the cell completes its growth, additional layers of cellulose fibres are deposited on the inner surface of the primary cell wall, which produces a secondary cell wall. The cell wall composition in vascular plants is approximately 30% cellulose, 30% hemicellulose, and 35% pectins, and 1–5% structural proteins, on a dry weight basis [2,3]. However, the precise proportions of these compounds may differ significantly at different stages of plant development. For example, grass coleoptiles consist of 60–70% hemicelluloses, 20–25% celluloses, and 10% pectin substances and the grass endosperm cell wall may contain up to as much as 85% hemicelluloses. By contrast, the secondary cell walls are generally more cellulose-rich [4,5,6,7].

*Brachypodium distachyon* (Brachypodium) belongs to the Pooideae subfamily and is a well-established model species for the grasses. It has several features and advantages that make it useful for gaining a better understanding of the genetic, cellular and molecular biology of temperate climate zone cereals and forage crops [8]. There are many studies, which are often connected with the chemical composition of the Brachypodium cell wall [9,10,11,12]. A comparative study of the primary cell wall in the seedlings of Brachypodium, barley and wheat demonstrated similar relative levels and developmental changes of hemicelluloses [10]. Analyses of the Brachypodium proteome facilitate better understanding of the enzymes that are involved in cell wall remodelling during seed development; such research is of great importance for gaining better understanding of these processes in grasses and for finding the key components that are responsible for the size and weight of grass grains [9]. However, there is a dearth of information about the localisation of specific cell wall components at different stages of Brachypodium development.

Here, we characterise the chemical composition of the cell walls in Brachypodium embryos and describe the differences in the number of nucleoli that were observed in the cell nuclei in different parts of an embryo. We used light and transmission electron microscopy (TEM), histological and immunolocalisation techniques to analyse the distribution of selected pectins, arabinogalactan proteins (AGP), extensins, and hemicelluloses in the cell walls, internal cell compartments, and on the embryo surface.

## 2. Results and Discussion

### 2.1. The Morphological and Histological Features of Brachypodium Embryos

**Figure 1:**
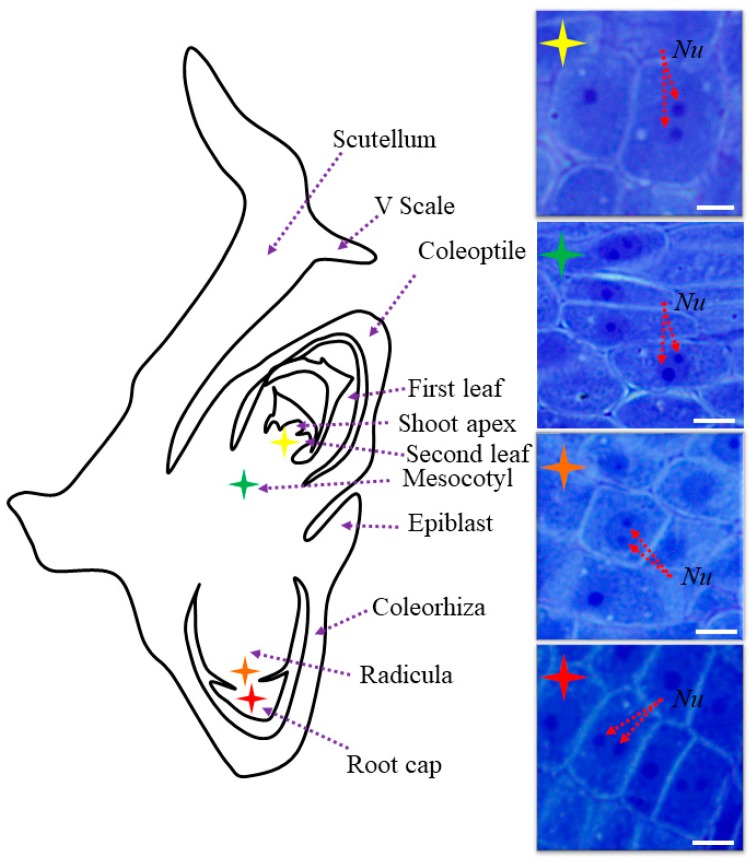
Schematic representation of Brachypodium embryo. Asterisks in different colours represent nuclei from histological sections of specified parts of the embryo. Red arrows mark two nucleoli. Purple arrows show respective parts of the embryo: V scale, coleoptile, first leaf, second leaf, shoot apex, mesocotyl, epiblast, coleorhiza, radicula, and root cap. Bars: 5 µm.

**Figure 2:**
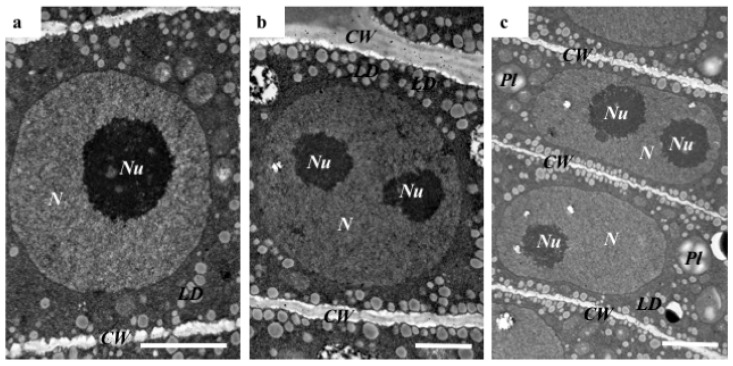
TEM (transmission electron microscopy) of radicula nuclei (**a**–**c**). Abbreviations: *CW*—cell wall, *LD*—lipid droplets, *N*—nucleus, *Nu*—nucleolus, *Pl*—plastid. Bars: (**a**) 3 µm; (**b**) 1.5 µm; and (**c**) 2.5 µm.

In their study, Wolny et al. [13] demonstrated that Brachypodium embryos are small in size, which makes their initial examination possible only by the use of a dissecting microscope. In this study, we distinguished the main parts of the embryo, such as scutellum, V scale, coleoptile, first and second leaf, shoot apex, mesocotyl, epiblast, radicula, root cap, and coleorhiza (Figure 1). The coleoptile and coleorhiza are two organs that are found exclusively in grass species [14]. A comparison of the cell nuclei in different parts of Brachypodium embryos demonstrated that the majority contained only one nucleolus (Figure 1). However, some cells of the shoot apex, mesocotyl, radicula and root cap were characterised by the presence of a round nuclei that contained two nucleoli (Figure 1; nucleoli indicated by red arrows). TEM analysis of the selected embryo parts confirmed these observations and demonstrated the presence of a centrally positioned nucleus with one or two large nucleoli as well as a high nucleus:cytoplasm ratio (Figure 2a,b). The cytoplasm of these cells was dense and contained lipid droplets and starch granules around the nucleus. Interestingly, we also found cells in the embryo with nuclei that were extended in their shape but that also contained two nucleoli (Figure 2c). The architecture of these cells is typical for the initial vascular tissue [15]. According to Verdeil et al. [16], pluripotent plant stem cells, which are located within the root and shoot meristems, are isodiametric, have a dense cytoplasm, a high nucleus:cytoplasm ratio, a fragmented vacuome, contain granules of starch, and have a spherically-shaped nucleus with one or two nucleoli. Both of the meristematic cells of the oil palm (*Elaeis guineensis*) [17], maize (*Zea mays*) [18], and onion (*Allium cepa*) [19] have similar characteristics to those described by Verdeil et al. [16], which appear to be universal for monocots.

### 2.2. AGP, Extensin, Pectin, and Hemicellulose Epitopes in Various Tissues and Organs of Brachypodium Embryos

**Figure 3:**
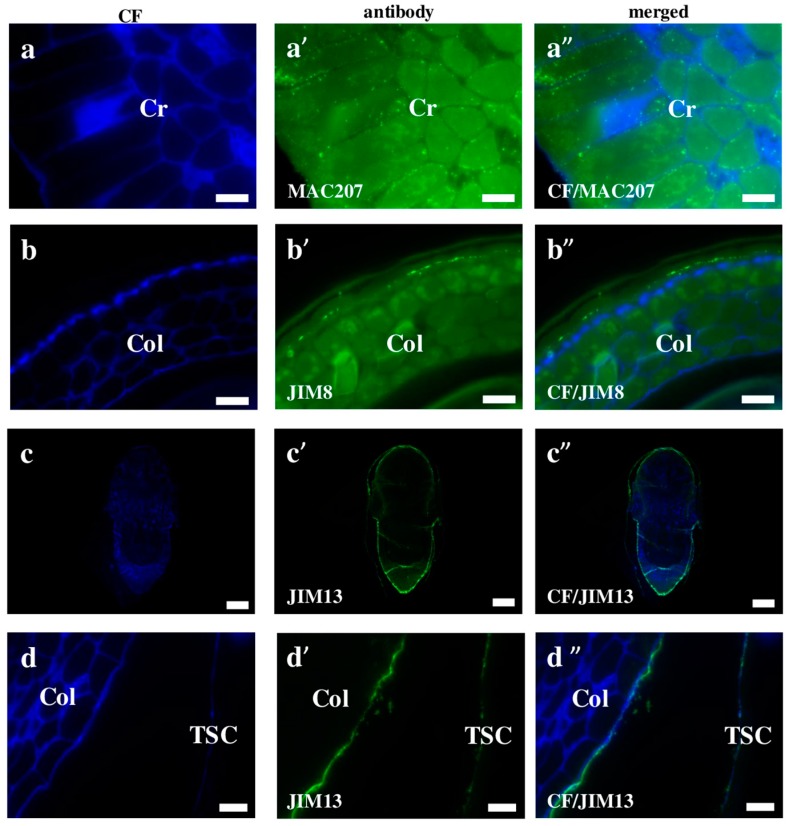
Immunolocalisation of arabinogalactan proteins in Brachypodium embryos. (**a**–**a”**) MAC207 in coleorhiza; (**b**–**b”**) JIM8 signal in coleoptile; (**c**–**c”**,**d**–**d”**) JIM13 in the entire embryo and the coleoptile with the seed coat, respectively. Abbreviations: CF—calcofluor, Cr—coleorhiza, Col—coleoptile, TSC—the seed coat. Bars: (**a**–**a”**,**b**–**b”**,**d**–**d”**) 10 µm; and (**c**–**c”**) 100 µm.

**Figure 4:**
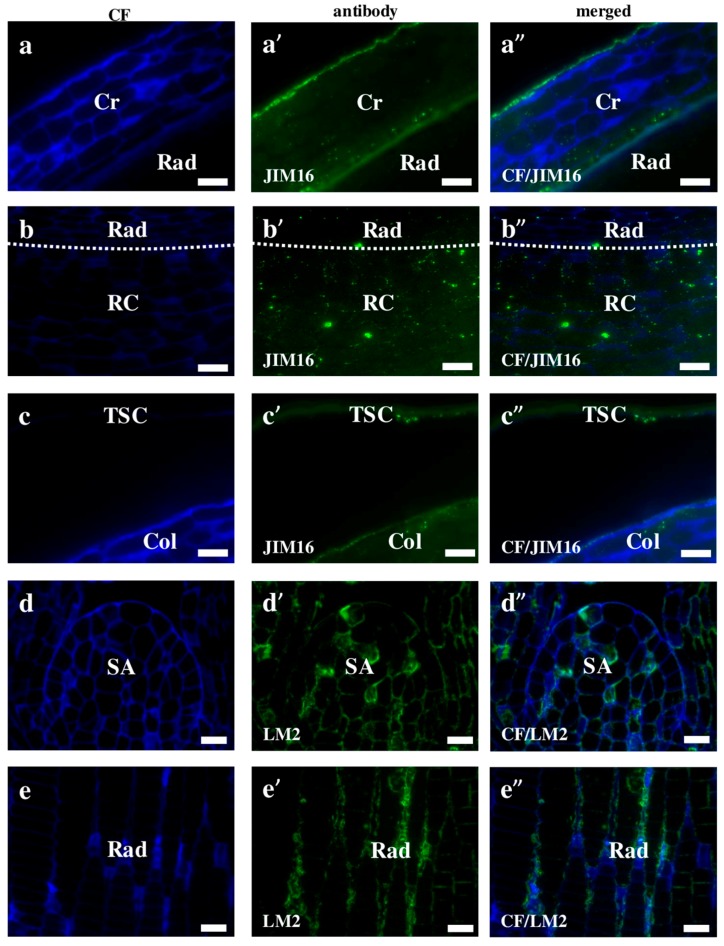
Immunolocalisation of arabinogalactan proteins in Brachypodium embryos. (**a**–**a”**,**b**–**b”**,**c**–**c”**) JIM16 with coleorhiza with radicula, radicula with root cap and coleoptile with the seed coat, respectively; Both (**d**–**d”**) shoot apex and (**e**–**e”**) radicula with LM2. Abbreviations: CF—calcofluor, Cr—coleorhiza, Col—coleoptile, Rad—radicula, RC—root cap, SA—shoot apex, and TSC—the seed coat. Bars: 10 µm.

AGPs distribution is dynamically regulated during the ontogenesis of a plant [20]. Although we demonstrated that AGPs have a diverse localisation in Brachypodium embryos, most of them are found on the cell surface. The MAC207 antibody had an affinity to all parts of the embryo and was localised in the internal cell compartments (Figure 3a–a”). JIM8, JIM13, and JIM16 were detected mainly in outer pereclinal walls of surface embryo tissues (Figure 3b–b”,c–c”,d–d” and Figure 4a–a”,c–c”). The JIM13 (Figure 3d–d”) and JIM16 (Figure 4c–c”) epitopes were components of the seed coat. Furthermore, the JIM16 epitope was localised in the internal cell compartments (Figure 4a–a”,b–b”). Compared to most of the analysed AGPs, the LM2 epitopes were found only in the cell wall compartments (Figure 4d–d”,e–e”). The possible functions of the AGPs on the embryo surface are to protect it from infections, contribute to plant-microbe interactions, seed germination and other important processes during plant development [20]. In their study of transcriptome and metabolome changes in *Arabidopsis thaliana* (Arabidopsis) that were connected with the seed dormancy and germination, Joosen et al. [21,22] showed that AGPs were more connected with the embryo cell walls. It is also known that AGPs are crucial in preventing infections in *Brassica napus* and *Pisum sativum* via the encystment of the *Aphanomces euteiches* zoospores, thereby inducing plant germination [23]. Van Hengel et al. [24] demonstrated that AtAGP30 is a non-classical AGP core protein in Arabidopsis, which had a root-tip specific expression in seedlings. This may imply its importance in root development or growth.

**Figure 5:**
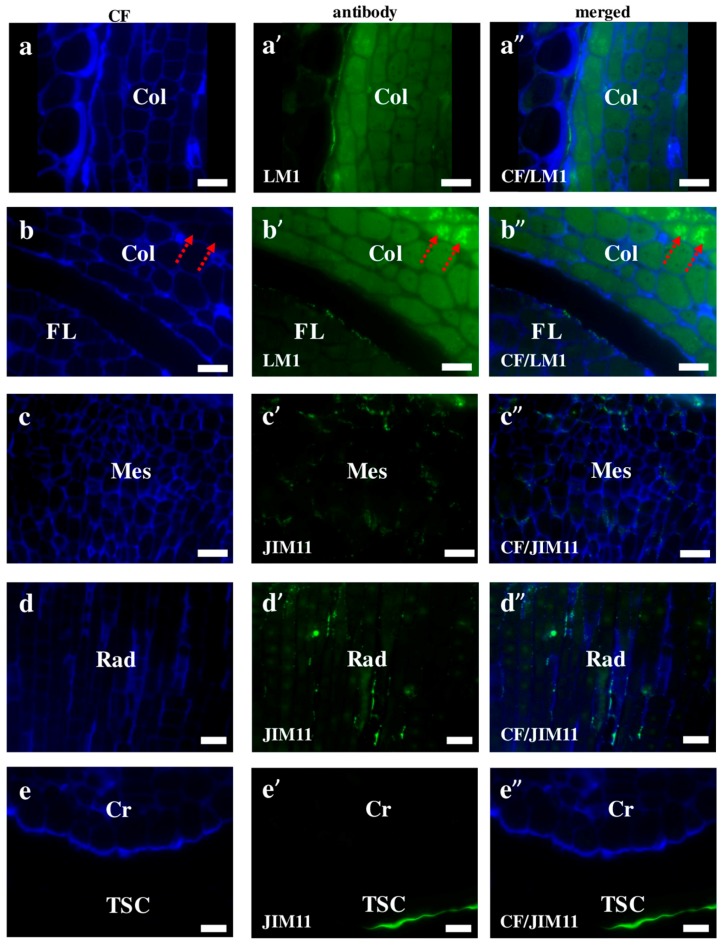
Immunolocalisation of extensins in Brachypodium embryos. Both (**a**–**a”**) coleoptile and (**b**–**b”**) coleoptile with first leaf with LM1; (**c**–**c”**,**d**–**d”**,**e**–**e”**) mesocotyl, radicula and coleorhiza (including the seed coat) with JIM11, respectively. Arrows show outer layers of coleoptile including protodermal cells (**b**–**b”**). Abbreviations: CF—calcofluor, Col—coleoptile, Cr—coleorhiza, FL—first leaf, Mes—mesocotyl, Rad—radicula, and TSC—the seed coat. Bars: 10 µm.

**Figure 6:**
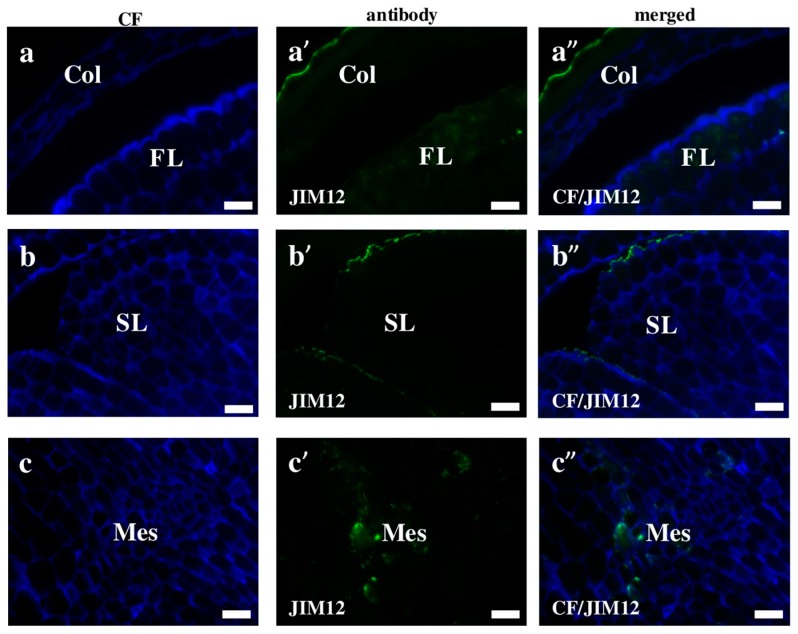
Immunolocalisation of extensins in Brachypodium embryos. (**a**–**a”**) coleoptile with first leaf; (**b**–**b”**) second leaf and (**c**–**c”**) mesocotyl. All with JIM12. Abbreviations: CF—calcofluor, Col—coleoptile, FL—first leaf, Mes—mesocotyl, and SL—second leaf. Bars: 10 µm.

Extensins are one of the major plant cell wall protein families found to be secreted into the cell wall. They are glycoproteins, in which about one-third of all amino acids is hydroxyproline with four monosaccharide arabinose residues attached. These compounds seem to play a strengthening and/or defensive role during the response to disease and wounding [25,26,27]. We observed that most of the Brachypodium extensins in this study had a surface localisation, which was similar to that of the AGPs. The signals of the LM1 antibodies were localised on the surface of the coleoptile and first leaf (Figure 5a–a”,b–b”). Furthermore, a high level of LM1 binding was observed in the internal compartments of the coleoptile epidermal cells (Figure 5b–b”; red arrows). The cell wall localisation was a specific feature of the JIM11 and JIM12 epitopes in the mesocotyl (Figure 5c–c” and Figure 6c–c”). Signals of the first antibodies were also observed in the cell walls of the radicula (Figure 5d–d”). Additionally, strong fluorescence signals of these antibodies were also found in the inner layer of the seed coat (Figure 5e–e”). The JIM12 extensin epitope had a surface distribution on the coleoptile, but no signals of these antibodies were observed in any part of the second leaf (Figure 6a–a”,b–b”). It is still not clear whether extensins have redundant or specific functions in the different cell types of various plant tissues. The inhibition of both extensin and AGP biosynthesis by 3,4-dehydro-l-proline impedes the development of embryogenic cells and decreases the rate of embryo germination [28]. This research demonstrated that extensins appear to play a vital role in the regeneration and germination of embryos during early plant development via somatic embryogenesis. It is also known that plants increase the amount of extensins when they are under aluminium stress. Sujkowska-Rybkowska et al. [29] suggested the importance of extensins in the aluminium resistance mechanisms and demonstrated an aluminium-induced extensin accumulation in the cell walls. Additionally, some extensins protect the plant cells from pathogen attacks [30]. An analysis of the extensin transcript levels in banana (*Musa* spp.) demonstrated that these proteins mainly appear in the root cap and meristematic cells after wounding or inoculation with *Fusarium oxysporum* [30]. The level of extensin transcription in intact plants were found to be up-regulated by wounding or inoculation with a pathogen, but down-regulated by a pathogen attack on wounded plants. In general, the connection of extensins with other components of the cell wall, as well as many of their functions during the development of the cell wall architecture are not yet fully understood [26]. It is likely that more extensive studies using mutants with the knocked down genes encoding extensins would shed more light on these matters.

**Figure 7:**
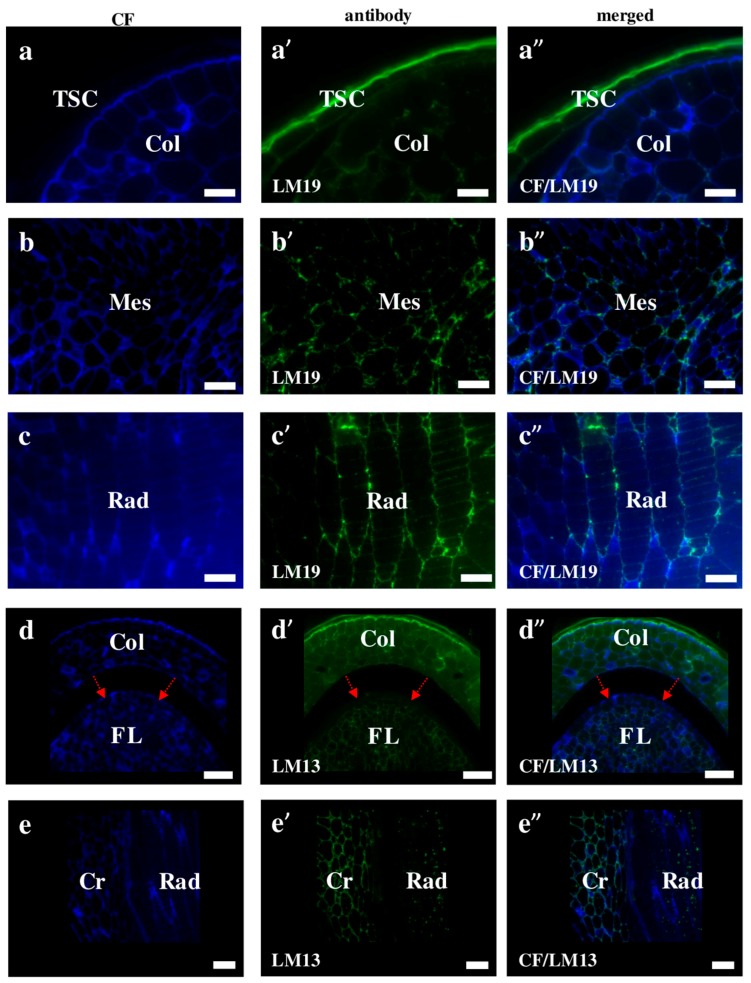
Immunolocalisation of pectins in Brachypodium embryos. (**a**–**a”**,**b**–**b”**,**c**–**c”**) Coleoptile with the seed coat, mesocotyl, and radicula are with LM19, respectively; Both (**d**–**d”**) coleoptile with first lead and (**e**–**e”**) coleorhiza with radicula with LM13. Arrows point to *FL* epidermal cells (**d**–**d”**). Abbreviations: CF—calcofluor, Col—coleoptile, Cr—coleorhiza, FL—first leaf, Mes—mesocotyl, Rad—radicula, and TSC—the seed coat. Bars: (**a**–**a”**,**b**–**b”**,**c**–**c”**) 10 µm; (**d**–**d”**,**e**–**e”**) 20 µm.

Pectins are high-molecular carbohydrates with d-galacturonic acid as their main structural component of that are found in the tissues of terrestrial plants and in some algae. In some plant tissues, for example in the white part of a citrus peel, the pectin content may reach up to 30% of dry weight, while, in others, it does not exceed a fraction of a percent [31,32]. Grasses, which contain a type II cell wall, are generally pectin poor [33]. The immunolocalisation of the homogalacturonan epitopes that recognised de-methyl-esterified pectin LM19 in Brachypodium were found on the outer surface of the seed coat (Figure 7a–a”). Moreover, LM19 was localised in different embryo parts, for example, in the cell wall of the mesocotyl and radicula, respectively (Figure 7b–b”,c–c”). The LM13 epitope could be a negative marker for the epidermal cells of the first leaf of a Brachypodium embryo. These antibodies gave signals in the cell walls in almost all parts of the embryo but were absent in the epidermal cells of the first leaf (Figure 7d–d”; red arrows). A different location of LM13 was observed in the coleorhiza and radicula cells. In the coleorhiza, this epitope was found in the cell walls, while in the radicula, it had an internal cell localisation (Figure 7e–e”). In Arabidopsis, the LM13 epitope is detectable in the epidermal cell walls of the younger, more flexible regions of the inflorescence stems and is almost absent in the base of the stem [34,35]. These authors demonstrated that the contribution of the arabinan structures to the cell wall mechanical properties influences the responsiveness to the mechanical stress. Therefore, the specific lack of the LM13 signals in the epidermal cells of the first leaf of Brachypodium embryo may be related to the absence of any mechanical stress in this part of an embryo.

**Figure 8:**
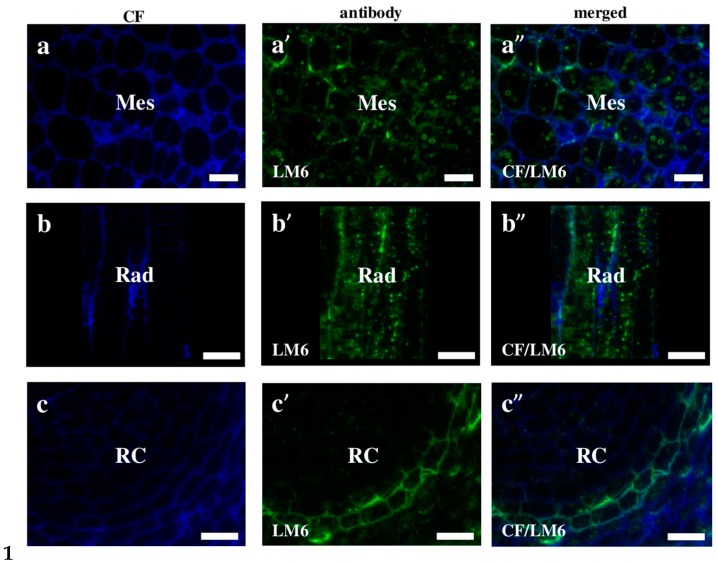
Immunolocalisation of pectins in Brachypodium embryos. (**a**–**a”**) Mesocotyl; (**b**–**b”**) radicula and (**c**–**c”**) root cap. All with LM6. Abbreviations: CF—calcofluor, Rad—radicula, RC*—*root cap, and Mes—mesocotyl. Bars: (**a**–**a”**,**b**–**b”**) 10 µm; (**c**–**c”**) 20 µm.

The fluorescence signals of LM6 against arabinans were found in both the cell wall and the internal cell compartment of the mesocotyl and radicula (Figure 8a–a”,b–b”). Furthermore, LM6 was localised on the external surface of the root cap but was absent in all other cells (Figure 8c–c”). As was demonstrated in Arabidopsis, the arabinans accumulated in developing and mature embryos but disappeared during germination and seedling establishment [36]. The early stages of Arabidopsis seed development showed a punctate distribution of this epitope compared to mature seeds, where LM6 labelling was very intense and distributed in the cell walls in the entire embryo. It was suggested that changes in the number and localisation of the arabinans might be connected with seedling germination. In barley, at the beginning of this process, the cell walls in the coleoptile have comparably high levels of arabinose-rich pectic polysaccharides, which decrease strongly during germination [37]. The abundance of the arabinans in the cell walls of seeds is very often a characteristic feature in a wide range of species such as almond (*Prunus dulcis*) [38], rapeseed (*Brassica napus*) [39], and honey locust (*Gleditsia triacanthos*) [40].

**Figure 9:**
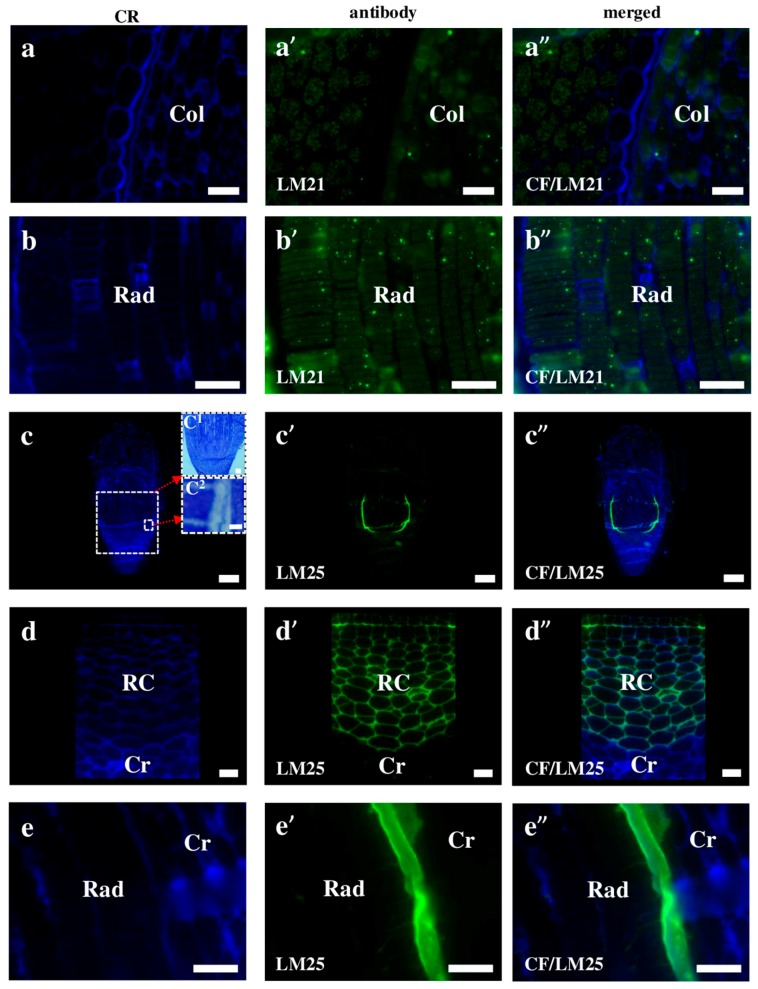
Immunolocalisation of hemicelluloses in Brachypodium embryos. (**a**–**a”**) coleoptile; (**b**–**b”**) radicula with LM21, respectively; (**c**–**c”**,**d**–**d”**,**e**–**e”**) the entire embryo, root cap with coleorhiza and radicula with coleorhiza with LM25, respectively. Arrows demonstrate insets: (**c^1^**,**c^2^**) histological sections of embryo, scale bars are 50 and 2 µm, respectively. Abbreviations: CF—calcofluor, Col—coleoptile, Cr—coleorhiza, Rad—radicula, and RC—root cap. Bars: (**a**–**a”**,**d**–**d”**,**e**–**e”**) 10 µm; (**b**–**b”**) 20 µm; (**c**–**c”**) 100 µm.

Hemicelluloses differ from cellulose in the composition of their monomers and their branched arrangement in molecules [7,41,42]. They are one of the components of the plastic matrix and impart additional strength to the cell wall, but do not hinder its growth [5]. Further, due to the ease of hydrolysis, they can also serve as reserve substances. In the Brachypodium embryo, the signals of the hemicellulose that recognise antibodies were localised in a different pattern. The signals of the LM21 antibodies were visualised in the internal cell compartments of all of the embryo parts, for example in the coleoptile and radicula (Figure 9a–a”,b–b”), while the LM25 antibodies were detected only in the radicula and root cap (Figure 9c–c”,d–d”,e–e”). Since it is difficult to determine whether the immunostaining is limited to only the cell wall, or whether it is also present outside of it using light microscopy, a TEM analysis was performed. Since this analysis was performed without immunostaining, it only showed the exact thickness of the wall (Figure S1). Thus, a calculation of the wall thickness (Figure 9c”) revealed that it was about 2 µm and that the thickness of the fluorescence “line” that was visible on Figure 9c’–c” was about 14.2 µm. This observation indicates that the hemicellulose epitopes were also outside the radicula protodermis, which can be explained by taking into account the development of the embryo, in which, during the final stages of development, detachment of the protective tissues such as the coleorhiza and coleoptile occur [43]. The localisation of some cell wall epitopes described in this work in the cellular compartments, indicate their extensive synthesis in the organelles, such as endoplasmic reticulum and Golgi apparatus dictiosomes, which are involved in this process [28,44,45]. It is worth noting that the same antibodies (LM21 and LM25) were localised in a different way in the embryogenic callus of Brachypodium [46]. As was demonstrated there, the LM21 antibodies only gave signals in the cell junctions of the embryogenic masses and in the parenchymatous cells. The signals of LM25 were found in every cell of both the embryogenic masses and parenchymatous cells.

In our current study, such a high deposition of xyloglucans seems to be important as a stock of elements for embryo development. Xyloglucans are known to differentially express throughout the embryo in Arabidopsis [47]. An analysis of Arabidopsis *xyl1* mutant phenotypes, which are associated with modifications of the composition of the endosperm cell wall demonstrated altered germination characteristics, such as shorter and thicker siliques, a reduced dormancy, and an increased tolerance to germination inhibitors and thermoinhibition. This study highlighted the role of xyloglucans during seed development and germination. Xyloglucans are likely to be very important components for cell adhesion and cell separation in fruit seed coat parenchyma [48]. Research on the tomato mutant *Cnr* (colourless non-ripening), which has a pleiotropic dominant mutation with reduced cell-to-cell adhesion, demonstrated that xyloglucan polymers (galactan and mannan) that are distributed more widely can be targeted to effect cell release from a parenchyma system. It was demonstrated that a monocotyledon palm *Euterpe oleraceae* contains a highly methoxylated homogalacturonan together with small amounts of a mannoglucan. Furthermore, a type II arabinogalactan was structurally characterised [49]. The experiments in non-lignified cell walls in different monocotyledon species, using the monoclonal antibody CCRC-M1 against fucosylated xyloglucans, demonstrated the presence of this epitope in non-lignified walls. A similar pattern was also found in the palm *Phoenix canariensis*. In Zingiberales, Commelinales, and Poales this epitope was found in the phloem walls, stomatal guard, subsidiary cells, and raphide idioblasts. However, it was not found in *Tradescantia virginiana* (Commelinaceae, Commelinales), or *Zea mays* (Poaceae, Poales). On the other hand, this epitope was observed in the phloem walls of two other Poaceae species, *Lolium multiflorum* and *L. perenne*. The authors highlighted the lack of knowledge about the functions of xyloglucans in monocotyledonous plants [50]. Moreover, work on Brachypodium revealed different proteome profiles that related to the different phases of grain development, and provided information of the reorganization of cell wall constituents that occurs during these stages [9,51]. Such results indicate that changes in cell wall chemical composition are important factor involved in its assembly and remodelling on different stages of development, plant tissues, and organs, both during embryonic and post-embryonic stages of development.

## 3. Materials and Methods

### 3.1. Plant Material and Histological Procedures

Approximately 15 mature embryos of Brachypodium (29 days after fertilization), reference genotype Bd21, were isolated from seeds that were collected from plants growing in pots with soil mixed with vermiculite (3:1) in a greenhouse. Plants were grown at 20 ± 1 °C, under a 16/8 h light/dark photoperiod. To ensure synchronised flowering, approximately two-week-old plants were subjected to vernalisation for two weeks at 4 °C. The embryos were fixed in a mixture of 4% (*w*/*v*) paraformaldehyde (PFA) and 1% (*v*/*v*) glutaraldehyde (GA) in phosphate-buffered saline (PBS, pH 7.0) overnight at 4 °C. Then, they were rinsed with PBS (3 × 15 min), dehydrated in an ascending ethanol series (10%, 30%, 50%, 70%, 90%, and 100%; 2 × 30 min in each) and gradually embedded in LR White resin [52]. The material was cut into 1.5-μm thick sections using an EMUC6 ultramicrotome (Leica Microsystems, Wetzlar, Germany). Sections were collected on microscopic slides covered with poly-l-lysine. For general histology, they were stained with 0.05% (aqueous solution) Toluidine Blue O (Sigma-Aldrich, St. Louis, MO, USA) for 5 min.

### 3.2. Immunocytochemistry

Sections were incubated in a blocking buffer containing 2% (*v*/*v*) foetal calf serum (FCS) and 2% (*w*/*v*) bovine serum albumin (BSA) in PBS (pH 7.2) for 30 min at room temperature (RT). Next, they were incubated with specific primary monoclonal antibodies (Table 1), diluted at a 1:20 ratio in a blocking buffer (RT, minimum 1.5 h), rinsed with the blocking buffer 3 × 10 min and then incubated at RT for at least 1.5 h with the secondary antibody (Alexa Fluor 488 goat anti-rat IgG, Jackson Immuno Research Laboratories, West Grove, PA, USA) diluted 1:100 in the blocking buffer as above. After washing with the blocking buffer and PBS (3 × 10 min each), the sections were stained with 0.01% calcofluor (Sigma-Aldrich, St. Louis, MO, USA) in PBS for 5 min; then slides were thoroughly rinsed with PBS and sterile distilled water (3 × 10 min each). The drained slides were mounted in a Fluoromount (Sigma-Aldrich) antifade medium. Negative controls were performed for each antibody that was used by omitting the primary antibodies. All of the images were taken using an Axio Imager Z2 (Zeiss, Oberkochen, Germany) epifluorescent microscope equipped with an AxioCam Mrm monochromatic camera (Zeiss) with the narrow-band filters for AlexaFluor 488 and DAPI. The negative control for all of the antibodies revealed the absence of any signals that were specific for these antibodies and was representative for all of the other antibodies that were used in this study (Figure S2).

### 3.3. TEM

The samples for TEM were fixed in 2.5% glutaraldehyde in a 0.1 M sodium phosphate buffer (pH 7.4) at 4 °C for 24 h. The embryos were postfixed in 1% OsO_4_ (osmium tetroxide) in a 0.1 M sodium phosphate buffer for two hours at 4 °C, rinsed in the same buffer, dehydrated in an ascending series of acetone, and gradually embedded in Epon 812 (Fullam, Latham, NY, USA) [66]. Semithin sections were stained with toluidine blue and examined with a bright-field microscope. Ultrathin (70 nm) sections were cut on a Leica ultracut UCT ultramicrotome and collected on copper grids (300 mesh, Electron Microscopy Science, Hatfield, PA, USA). Sections were stained with uranyl acetate and lead citrate and examined with a Hitachi H500 TEM (Hitachi, Tokyo, Japan) at 75 kV.

## 4. Conclusions

In this work we traced the distribution of various cell wall epitopes in the Brachypodium zygotic embryo in mature stages of development. We have demonstrated that:

1. Among analysed AGP epitopes JIM13, JIM8, and LM2 were localised in cell walls of the embryo surface tissues, as well as some of them in the seed coat; and MAC207 and JIM16 were detected in cytoplasmic compartments.
2. Extensins are localised in the outer periclinal walls of embryo surface tissues. Our results suggest that in Brachypodium, the antibody LM13 can be used as a negative marker of epidermal cells of the embryonic leaf and that the LM25 antibody can be used as the positive marker of embryonic root epidermis cells, as well as the root cap in Brachypodium. These results demonstrate that the distribution of analysed cell wall epitopes in mature zygotic embryos of Brachypodium is tissue- and organ specific.

Without a doubt, the precise dissection of all functions of the cell wall in Brachypodium embryos requires the use of specific mutants, whose availability is limited. However, this problem should soon be overcome by the rapidly-developing techniques of site-directed mutagenesis, such as CRISPR/Cas9 that are already available for Brachypodium [67].

## Figures and Tables

**Table 1:** The antibodies used for immunocytochemistry, the epitopes they recognise, and relevant references.

Antibody	Epitope	References
	*Arabinogalactan proteins (AGPs)*	
MAC207	β-GlcA1->3αGalA1->2Rha	[53,54,55,56]
JIM8	Arabinogalactan	[57]
JIM13	βGlcA1->3αGalA1->2Rha	[55,56,58]
JIM16	AGP glycan	[55,56,58]
LM2	β-linked GlcA	[56,59]
	*Extensins*	
LM1	Extensin	[60]
JIM11	Extensin	[55,61]
JIM12	Extensin	[61]
	*Pectins*	
LM19	α-GalA(1-4)α-GalA(1-4)α-GalA(1-4)α-GalA	[35]
LM13	α-1,5-arabinan	[62]
LM16	Processed arabinan—rhamnogalacturonan (RG)-I domain	[35]
LM6	αAra1-5αAra1-5αAra1-5αAra1-5Ara	[63]
	*Hemicelluloses*	
LM21	β-linked mannan polysaccharides of plant cell walls	[64]
LM25	Xyloglucan	[65]

## Supplementary Materials

The following are available online at http://www.mdpi.com/1422-0067/19/3/725/s1.

## Abbreviations

AGP	Arabinogalactan protein
BSA	Bovine serum albumin
CRISPR	Clustered regularly-interspaced short palindromic repeats
FCS	Foetal calf serum
GA	Glutaraldehyde
PBS	Phosphate-buffered saline
PFA	Paraformaldehyde
RT	Room temperature
TEM	Transmission electron microscope
