# Supplementary material for: Organ and Tissue-Specific Localisation of Selected Cell Wall Epitopes in the Zygotic Embryo of Brachypodium distachyon
Source: Int J Mol Sci. 2018 Mar 3;19(3):725. doi: 10.3390/ijms19030725 (PMC5877586; doi:10.3390/ijms19030725)
Supplement: Supplementary file 1 [file ijms-19-00725-s001.pdf]

Supplementary Materials: Organ and Tissue-Specific Localisation of Selected Cell Wall Epitopes in the Zygotic Embryo of *Brachypodium distachyon*

Alexander Betekhtin, Anna Milewska-Hendel, Joanna Lusinska, Lukasz Chajec, Ewa Kurczynska and Robert Hasterok

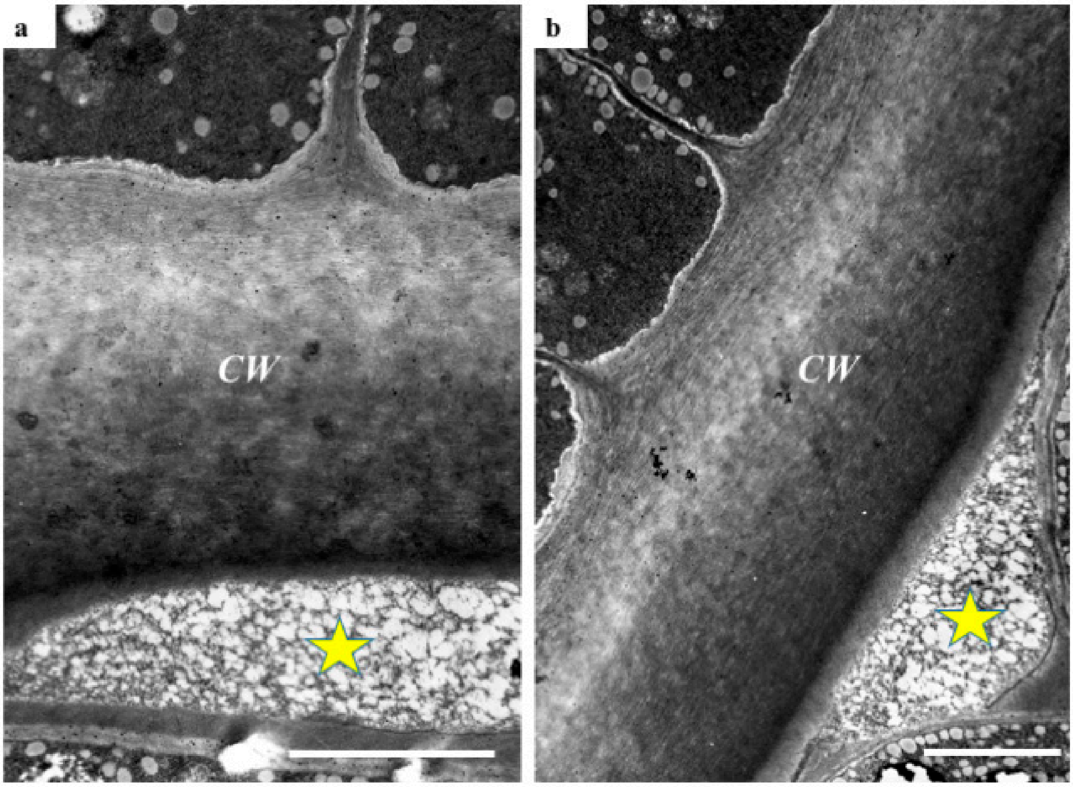

Figure S1. TEM of the radicula epidermis (**a**,**b**). Yellow stars showing the space with fibrillary component between radicula and coleorhiza. Abbreviations: *CW*—cell wall. Bars: (**a**) 3 μm; (**b**) 4.5 μm.

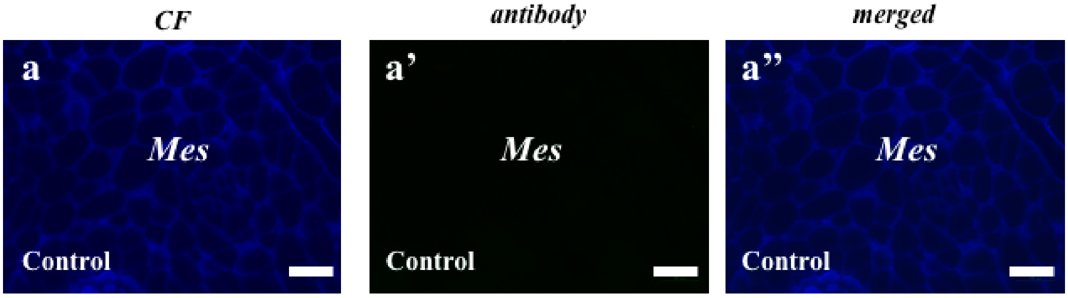

Figure S2. Negative control. (**a**–**a″**) no antibody fluorescent signals are present. Abbreviations: *CF*—calcofluor, *Mes*—mesocotyl. Bars: 10 μm.
